# Affective tactile stimulation ameliorates chronic pain and comorbid anxiety by modulating hippocampal CA1 oscillations and functional connectivity

**DOI:** 10.3389/fnmol.2026.1844176

**Published:** 2026-07-29

**Authors:** Li Zeng, YuJia Jin, Lei Chen, YiTian Lai, YuanQian Zhu, YuQiao Zhang, Xiang Feng, JiangShan Li, Wu Li

**Affiliations:** 1 School of Acupuncture-moxibustion Tuina and Rehabilitation, Hunan University of Chinese Medicine, Changsha, China; 2 The Second Hospital of Hunan University of Chinese Medicine, Changsha, China

**Keywords:** affective tactile stimulation, chronic pain with anxiety, fMRI, hippocampal CA1, neural oscillations

## Abstract

Chronic pain is a multidimensional experience influenced by biological, psychological, and social factors. It frequently culminates in a reciprocal “pain-emotion” vicious cycle involving comorbid anxiety and depression. This study investigated the therapeutic potential of affective tactile stimulation in a rat model of chronic inflammatory pain with anxiety, focusing on the afferent pathways and the regulatory role of the hippocampal CA1 region. Through behavioral assays and optogenetic interventions, we demonstrated that affective tactile stimulation partially ameliorates pain hypersensitivity, exerts a mild modulatory effect on peripheral inflammatory responses, and effectively alleviates anxiety-like behaviors. Mechanistically, affective tactile stimulation appears to integrate sensory information within the tactile cortex to modulate neural activity in the hippocampal CA1, a key regulatory hub. Enhanced CA1 neuronal activity was found to correlate closely with symptom improvement. Furthermore, our results suggest that oscillatory enhancement in the theta and delta bands, alongside functional connectivity remodeling, might play a critical role in these effects. These findings provide a neurobiological foundation for the clinical application of affective tactile stimulation in managing chronic inflammatory pain and associated affective disorders.

## 1 Introduction

Chronic inflammatory pain is defined as a persistent, subjective sensation that lasts beyond the expected healing period of 3 months. It is shaped by complex bio-psycho-social interactions (Cohen et al., 2021) and is frequently exacerbated by anxiety and depression, forming a debilitating “pain-emotion” feedback loop (Bushnell et al., 2013; Spohn et al., 2013). Epidemiological data indicate that approximately 50% of chronic pain patients suffer from comorbid anxiety disorders (Feingold et al., 2017), which severely compromise their quality of life and present a significant challenge for clinical management.

To address this complex interplay between chronic pain and emotional distress, sensory modulation has emerged as a promising therapeutic strategy. Beyond its discriminative capacity, affective tactile stimulation possesses well-documented analgesic properties and plays a critical role in emotional regulation. Pleasurable social contact, as a common prosocial behavior, is essential for establishing and maintaining social bonds (Reddan et al., 2020; Hu and Hu, 2022). In clinical care, appropriate affective tactile stimulation is a vital therapeutic factor; for instance, it is frequently employed to alleviate emotional distress such as depression and anxiety in patients (Guo et al., 2020; Watt et al., 2021). However, the neural circuits governing the regulation of chronic inflammatory pain with anxiety remain poorly defined. Specifically, elucidating how affective tactile stimulation mitigates mood disorders through peripheral-to-central signaling is an urgent scientific question.

The onset of affective tactile stimulation starts from the skin of the patient as the focus point to activate the receptors (Taneja et al., 2021), and different afferent fibers, including Aβ, Aδ, and C fibers, are activated at different rates, exerting both peripheral and central effects, and ultimately acting locally on the lesion (Handler and Ginty, 2021). Affective tactile stimulation acting comfort receptors are mainly low-threshold mechanosensory receptors (LTMR) (Meijer et al., 2022; Schirmer et al., 2023). Taking the skin as an example, nerve fibers from the dorsal root ganglia (DRG) are distributed in the subcutaneous area and are matched with various types of mechanoreceptors that respond to skin deformations in specific ways and transmit these stimuli to higher brain structures (Roudaut et al., 2012). Light touch receptors need to respond to small forces, and LTMRs are mainly composed of Merkel cells, specialized cells in hairless skin, and various types of nerve ending vesicles, such as Ruffini endings, Meissner corpuscles, Pacinian corpuscles, and a variety of longitudinal and circumferential needle-like low-threshold mechanoreceptors that surround hair follicles (Stecina et al., 2013; Dunkley et al., 2020; García-Mesa et al., 2022). Meanwhile, Aβ, Aδ, and C fibers propagate to the spinal cord at markedly different rates, and slow cutaneous tactile sensations particularly activate non-peptidergic C fibers.

Both chronic and evoked pain affect attentional performance (Moore et al., 2019), and distraction leads to reduced pain sensitivity (Hoegh et al., 2019). Thus, affective tactile stimulation enhances extrinsic sensory input, influencing functional adaptation to pain and producing the affective emotion of pain relief. Another cognitive function associated with pain is the memory (Johnston et al., 2012). Memory consists of a series of storage systems in which information flows from the environment into a series of temporary sensory buffers that are essentially part of the perceptual process, which includes the perception of touch, and then passes to short-term memory stores of limited capacity, which are then input into long-term memory (Baddeley, 2010). As a result of the involvement of memory, the sense of tactile interaction gives rise to affective feelings.

As a major structure of the limbic system, the hippocampus plays a key role in pain perception and chronic inflammatory pain (Liu and Chen, 2009; Baliki and Apkarian, 2015). Persistent pain inhibits hippocampal neurogenesis, synaptic plasticity and c-Fos expression, reduces hippocampal volume, and disrupts functional connectivity within the hippocampus (Khanna et al., 2004; Mutso et al., 2012; Mutso et al., 2014). Functionally, the hippocampus is segmented along a dorso-ventral axis, with the dorsal region involved in episodic memory and the ventral region involved in emotional behavior (Fanselow and Dong, 2010; Strange et al., 2014). In rodents, inhibition of dorsal hippocampal activity is the root cause of cognitive deficits associated with chronic inflammatory pain (Cardoso-Cruz et al., 2018), whilst the ventral hippocampus is more involved in the perception and emotional aspects of pain (Zheng et al., 2017; Jiang et al., 2018; Ma et al., 2019; Wang et al., 2024).

Affective tactile stimulation may influence pain-related affective and sensory processing through low-threshold mechanoreceptor-mediated tactile pathways. Rather than directly transforming pain into a pleasurable emotion, such stimulation may provide non-noxious somatosensory input that modulates pain-related behavioral responses and brain activity (Saffarpour and Nasirinezhad, 2021; Huzard et al., 2022; Schaefer et al., 2022; Della Longa et al., 2025). Based on the known role of tactile afferent pathways and hippocampal CA1 activity in pain- and anxiety-related regulation, we hypothesized that affective tactile stimulation may alleviate chronic inflammatory pain-associated anxiety by modulating CA1 oscillatory activity and functional connectivity.

## 2 Materials and methods

### 2.1 Experimental apparatus and reagents

Affective tactile stimulation tactile stimulator (Supplementary Figure 1), mechanical pain threshold measuring instrument (Beijing Zhongshi DiChuang Science and Technology Development Co., Ltd., model ZS-CTY), foot swelling measuring instrument (Ugo Basile, Italy), small animal respiratory anesthesia machine (Reward Life Science and Technology Co., Ltd.), slicing machine (Leica Instruments Shanghai Co., Ltd., RM2016), inverted fluorescence microscope (Zeiss, Germany), 4% formaldehyde solution (Biosharp), Complete Freund’s Adjuvant (Sigma, United States), isoflurane inhalation anesthetics (Reward Life Science and Technology Co., Ltd.), and duloxetine (Shanghai Jizi Biochemical Technology Co., Ltd.), and behavioral science (Beijing Zongshi Dichuang Technology Development Co., Ltd.), 7.0T Biospec 70/20 USR Small Animal MR System (Bruker, Germany), Cerebus 128-channel Neural Signal Acquisition System for Electrophysiology (Blackrock, United States), Intelligent Limited Optogenetic System [Inper Fluorbo(Ltd.)], Multi-Channel Fiber Optic Recording System [Inper Fluorobot (Hangzhou) Technology Co., Ltd.], rAAV-hSyn-hChR2(H134R)-EYFP (Brinkus (Shenzhen) Biotechnology Co., Ltd. Reference No. BC-0346), rAAV-hSyn-eNpHR3.0-mCherry [Brinkus (Shenzhen) Biotechnology Co., Ltd. Reference No. BC-0119], rAAV-hSyn-GCaMP6f [Brinkers (Shenzhen) Biotechnology Co. Reference No. BC-0079].

### 2.2 Animals

Source of animals: provided to the Animal Experiment Center of Hunan University of Chinese Medicine by Hunan Slaughter Kingda Laboratory Animal Co. Male SPF-grade Sprague-Dawley (SD) rats (4–5 weeks old, weighing 120–150 g). Animals were housed individually under a 12 h light/dark cycle (24–26°C, 50–70% humidity) with *ad libitum* access to food and water. All animals were allowed to acclimatize to their environment for at least 1 week prior to any experimental procedures.

### 2.3 Experimental design and timeline

**FIGURE 1:**
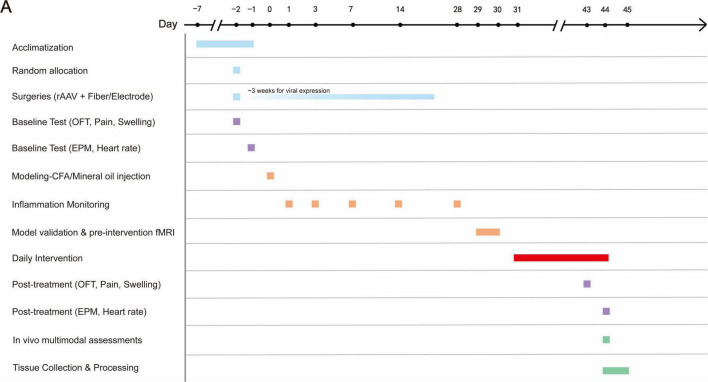
Overview of the experimental timeline. The timeline illustrates the four phases of the study. Phase 1 from day-7 to -1 includes baseline measurements and stereotaxic surgeries. Phase 2 spans days 0–30 and covers chronic pain induction via Complete Freund’s Adjuvant injection, with mineral oil utilized as a vehicle control, followed by subsequent model validation. Phase 3 details the 14-day therapeutic intervention period from days 31 to 44. Phase 4, from days 43 to 45, outlines comprehensive post-intervention evaluations and tissue harvesting.

The experimental timeline consisted of four phases (Figure 1):

Phase 1: Preparation and Baseline (day-7–1). Following a 1-week acclimatization with daily handling, animals were randomized on day-2. Stereotaxic surgeries (rAAV injection and optic fiber/electrode implantation in the hippocampal CA1) were performed, allowing ∼3 weeks for viral expression. Baseline OFT, mechanical pain threshold, and paw swelling were assessed on day-2, followed by baseline EPM and heart rate on day-1.

Phase 2: Modeling and Validation (days 0–30). On day 0, animals received a subcutaneous injection of Complete Freund’s Adjuvant (CFA) into the right hind paw to induce chronic pain, or mineral oil as a vehicle control. Inflammatory progression was monitored on days 1, 3, 7, 14, and 28. Successful establishment of the “chronic pain with anxiety” model and pre-intervention fMRI scans were completed on days 29–30.

Phase 3: Intervention (days 31–44). Animals received 14 consecutive days of daily interventions: 20 min of mechanical stroking (affective tactile stimulation group) or oral duloxetine (pharmacological control group).

Phase 4: Evaluation and Tissue Collection (days 43–45). Post-treatment OFT, pain thresholds, and paw swelling were evaluated on day 43. On day 44, EPM, heart rate, and *in vivo* multimodal assessments (fMRI, optogenetics, fiber photometry) were conducted, and a subset of rats was euthanized for hind paw H&E staining. On day 45, the remaining rats were transcardially perfused (4% PFA) for brain extraction and downstream molecular/fluorescence assays.

### 2.4 Establishment of CFA-induced model and experimental design

To investigate the multidimensional modulatory effects of affective tactile stimulation, rats were randomly allocated into three independent experimental cohorts: a behavioral and morphological cohort (*n* = 6 per group), an optogenetic cohort (*n* = 4 per group), and an electrophysiological cohort (*n* = 4 per group).

Under isoflurane anesthesia (induction: 2–3%; maintenance: 1.5–2%), rats received a subcutaneous injection of 100 μL complete Freund’s adjuvant (CFA) into the right hind paw to establish a chronic inflammatory pain model accompanied by anxiety-like behaviors. Control animals received an equivalent volume of mineral oil injected at the same anatomical site. Model establishment was validated on day 28 post-injection by the presence of local paw swelling, a significant reduction in mechanical withdrawal threshold, and anxiety-like behaviors assessed using the open field test and elevated plus maze test (Steimer, 2011; Wu et al., 2017).

### 2.5 Intervention protocols

The intervention was administered for 14 consecutive days from day 31 to day 44 post-modeling.

#### 2.5.1 Affective tactile stimulation

To ensure that the mechanical stimulation was non-aversive, a progressive habituation protocol was implemented during the 1-week acclimatization period prior to CFA modeling. Rats were placed in the stimulation apparatus for 10 min daily and initially habituated to environmental stimuli associated with device operation, including motor-generated noise and vibration, before exposure to gentle static contact stimulation.

To minimize operator-dependent variability and ensure experimental standardization, a custom-designed automated tactile stimulation device was employed. Combining this with previous animal experiments on affective tactile stimulation, gentle stroking has been defined as a form of affective sensation (Liu et al., 2022). The applied force, contact area, rotational speed, and daily duration of the stimulation were established based on pressure intensity parameters from prior studies and subsequent preliminary experiments (Jiang, 2022). These baseline data were converted into light pressure levels appropriate for rodent models. This optimization aimed to deliver stable and completely pain-free mechanical stimulation to the forehead, strictly avoiding the induction of stress-related behaviors such as avoidance, struggling, vocalization, or rigidity. The stimulation parameters were strictly maintained as follows: an applied force of 0.3 kg, a contact area of 2 cm^2^, and a rotational frequency of 80 rpm. Mechanical tactile stimulation was delivered to the forehead region of the rats once daily, with each session lasting 20 min (Chen et al., 2025).

During both the habituation period and the subsequent 14-day intervention phase, the rats exhibited no observable escape, struggling, or defensive behaviors. Instead, the animals rapidly adopted resting postures, serving as behavioral indicators of relaxation and acceptance of the stimulation procedure.

#### 2.5.2 Pharmacological control

Rats in the duloxetine-treated group received duloxetine (10 mg/kg) via oral gavage once daily for 14 consecutive days (Müller et al., 2008).

### 2.6 Behavioral and physiological assessments

All behavioral assessments were performed by experimenters blinded to group allocation. Prior to testing, animals were allowed to acclimatize to the experimental environment for at least 30 min.

#### 2.6.1 Mechanical withdrawal threshold and paw edema assessment

Mechanical withdrawal thresholds were measured using a mechanical pain threshold analyzer. Vertical pressure was applied to the plantar surface of the right hind paw, and the pressure value eliciting paw withdrawal or vocalization was recorded. Each measurement was repeated three times, and the average value was calculated. Paw edema was quantitatively evaluated using a paw volume meter by immersing the hind paw to a level below the ankle joint.

#### 2.6.2 Open field test (OFT)

To minimize stress and adapt the animals to human contact, all rats were handled for approximately 2 min daily for five consecutive days prior to the behavioral assays. Spontaneous locomotor activity and anxiety-like behaviors were evaluated using an open-field arena (50 × 50 × 50 cm) constructed of polyvinyl chloride (PVC). A 25 × 25 cm concentric square was virtually defined as the central zone. One day prior to the formal testing, the rats were allowed to freely explore the arena for 15 min to habituate to the novel environment. During the actual testing days, the animals were transferred from the vivarium to the behavioral testing room and allowed to acclimatize for at least 30 min. During the test, each rat was placed in the arena and allowed to freely explore for 5 min, immediately followed by a 5-min continuous recording phase. Following the test, the animals were returned to their home cages. Behavioral parameters, including the time spent in the central zone, average velocity, and total distance traveled, were quantified and analyzed.

#### 2.6.3 Elevated plus maze (EPM)

Anxiety-like behavior was further assessed using an elevated plus maze. The apparatus was elevated 60 cm above the floor and consisted of two open arms (35 × 6 cm), closed arms (35 × 6 cm), and a central platform (6 × 6 cm). Prior to the testing, the behavioral tracking software (Tracking Master V4.0) and recording equipment were initialized and calibrated to optimal settings. Each rat was gently placed on the central platform facing an open arm, and its exploratory behavior was continuously recorded for 5 min. Behavioral parameters, including the number of open-arm entries, the time spent in the open arms, and the total distance traveled, were subsequently quantified and analyzed.

#### 2.6.4 Heart rate monitoring

Rats were anaesthetized with isoflurane and maintained at 37°C on a heating pad throughout the recording session. Physiological signals were continuously acquired via subcutaneous electrodes connected to an MP160 data acquisition system and analyzed using AcqKnowledge software.

### 2.7 Stereotaxic surgery, optogenetics, and fiber photometry

Rats were anaesthetized with isoflurane and positioned in a stereotaxic frame. Following exposure of the skull, the hippocampal CA1 region was targeted using the following stereotaxic coordinates relative to bregma: anteroposterior (AP), −3.6 mm; mediolateral (ML), ± 2.0 mm; dorsoventral (DV), −2.8 mm. A total volume of 350 nL viral vector was bilaterally delivered using a microinfusion pump at a rate of 30 nL/min into the hippocampal CA1 region.

#### 2.7.1 Optogenetic manipulation

rAAV-hSyn-hChR2(H134R)-EYFP or rAAV-hSyn-eNpHR3.0-mCherry viral vectors were injected into the hippocampal CA1 region. Three weeks post-injection, optical fibers were implanted unilaterally above the target region, primarily targeting the right CA1 region. Optogenetic manipulation was performed using an intelligent optogenetic stimulation system.

For ChR2-mediated photoactivation, 465-nm blue light was delivered using a square-wave pulsed stimulation protocol. Blue-light pulses were delivered at 20 Hz with a 5-ms pulse width during the behavioral testing window. The device power was set within the calibrated output range of 70%. Each stimulation sweep lasted 300 s. For eNpHR3.0-mediated photoinhibition, 589-nm yellow light was delivered using a constant-output protocol rather than a pulsed stimulation protocol. The stimulation waveform was set to constant illumination with 0-Hz frequency and 0-ms pulse width. Yellow light was delivered at 65% device power in sustained 300 s illumination epochs during the behavioral testing window.

Optogenetic stimulation was time-locked to behavioral testing. For open-field testing and paw withdrawal threshold assessment, behavioral measurements were first obtained during a baseline Laser-OFF period, followed by a Laser-ON testing period during which the corresponding optogenetic stimulation protocol was applied (Supplementary Figure 2).

#### 2.7.2 Optical fiber calcium imaging

To monitor population calcium dynamics in the hippocampus, a viral vector encoding a calcium indicator under a pan-neuronal promoter, rAAV-hSyn-GCaMP6f, was injected into the CA1 region. Three weeks post-surgery, following recovery and behavioral habituation, bulk calcium fluorescence signals (ΔF/F) were recorded in freely moving animals using a multichannel fiber photometry system. The recording side was selected to correspond to the right CA1 cluster identified in the fMRI analysis.

To ensure precise synchronization between the physiological recordings (GCaMP calcium signals) and the behavioral intervention, temporal alignment was strictly achieved through a protocol of real-time manual event logging, each individual tactile contact/stroking episode was defined as one event. Specifically, trained experimenters visually monitored the exact moment of affective tactile stimulus delivery and synchronously recorded the precise onset and offset times directly within the data acquisition software to generate reliable event markers. During the subsequent offline analysis, these carefully documented timestamps were utilized to extract the affective tactile stimulation-related calcium transients, thereby establishing robust time-locking for all downstream data processing. Finally, the area under the curve (AUC) of the ΔF/F signal during the defined post-stimulus window was calculated for quantitative statistical comparisons (Supplementary Figure 3).

### 2.8 fMRI acquisition and preprocessing

Images were acquired using a 7.0 T small-animal MRI system. T2-weighted images were obtained using a fast spin-echo sequence with the following parameters: repetition time (TR) = 4,000 ms, echo time (TE) = 36 ms, slice thickness = 0.8 mm, and 26 slices. Functional images were acquired using an echo-planar imaging (EPI) sequence with the following parameters: TR = 2,000 ms, TE = 14 ms, flip angle = 90°, and matrix size = 72 × 72. Image preprocessing was performed using DPABI and SPM12 software packages, including slice timing correction, head motion correction, spatial normalization, detrending, and temporal band-pass filtering (0.01–0.08 Hz). Data exhibiting head motion exceeding 0.3 mm translation or 3° rotation were excluded from further analysis. Regional homogeneity (ReHo) and amplitude of low-frequency fluctuations (ALFF) were subsequently calculated. Whole-brain functional connectivity (FC) analysis was then performed based on 152 gray matter regions of interest (ROIs) defined according to the SIGMA atlas.

### 2.9 Local field potential

Local field potential (LFP) signals were recorded from the hippocampal CA1 region using a Blackrock Cerebus neural signal acquisition system. The filtered continuous NS2 data were used for LFP analysis. Signals were sampled at 1,000 Hz from four channels and stored in microvolts (μV). the continuous LFP signals were band-pass filtered from 250 Hz low-pass filters. An approximately 300-s recording window was used for spectral analysis. LFP spectral analysis was performed using NeuroExplorer. Time-frequency analysis was conducted using a multiple-taper spectrogram with a time-bandwidth product of 3 and 5 tapers. The first analysis window started at 1 s, and 300 sliding windows were used with an absolute shift of 1 s. Spectrogram values were normalized as percentage of total PSD and displayed over 0–30 Hz.

PSD analysis was performed using a multiple-taper method with a time-bandwidth product of 3 and 5 tapers. Window overlap was set to 1%, incomplete windows were discarded, and selected data points were concatenated before analysis. PSD values were exported as log-transformed PSD in decibels (dB). PSD was calculated over 0–80 Hz. Delta and theta band activities were quantified from the exported spectral values over 1–4 and 4–8 Hz, respectively.

### 2.10 Hematoxylin and eosin staining

At the end of the 44-day experimental period, rats were deeply anaesthetized with sodium pentobarbital, and tissue samples were collected from the plantar region of the right hind paw. The tissues were fixed in 4% paraformaldehyde for 24 h, decalcified in 5% nitric acid for 5 days, neutralized with 5% sodium sulfate solution, and subsequently processed for routine paraffin embedding. Paraffin-embedded tissues were sectioned at a thickness of 5 μm.

Following deparaffinisation and rehydration, sections were subjected to hematoxylin and eosin (H&E) staining and mounted for histological examination. Histopathological changes, including inflammatory cell infiltration and alterations in muscle fiber morphology, were observed under a light microscope.

### 2.11 Statistical analysis

Statistical analyses were performed using SPSS version 25.0 software. Normally distributed data are presented as mean ± standard deviation (mean ± SD). Repeated-measures analysis of variance (repeated-measures ANOVA) was used for longitudinal data involving time-dependent variables, such as mechanical withdrawal thresholds and paw edema measured at multiple time points. Comparisons among multiple groups at a single time point were performed using one-way ANOVA, whereas comparisons between two groups were conducted using the independent-samples *t*-test.

Non-normally distributed data are presented as median with interquartile range (IQR) and were analyzed using the Mann–Whitney U test or Wilcoxon signed-rank test, as appropriate.

For fMRI data, voxel-wise analysis was performed using a mixed-effects model. Statistical significance was defined using a voxel-level threshold of *P* < 0.001 and a cluster-level threshold of *P* < 0.05 following Gaussian random field (GRF) correction for multiple comparisons. A two-tailed *P* < 0.05 was considered statistically significant for all analyses.

## 3 Results

### 3.1 Affective tactile stimulation exerts comprehensive bidirectional modulation on comorbid chronic pain and anxiety

**FIGURE 2:**
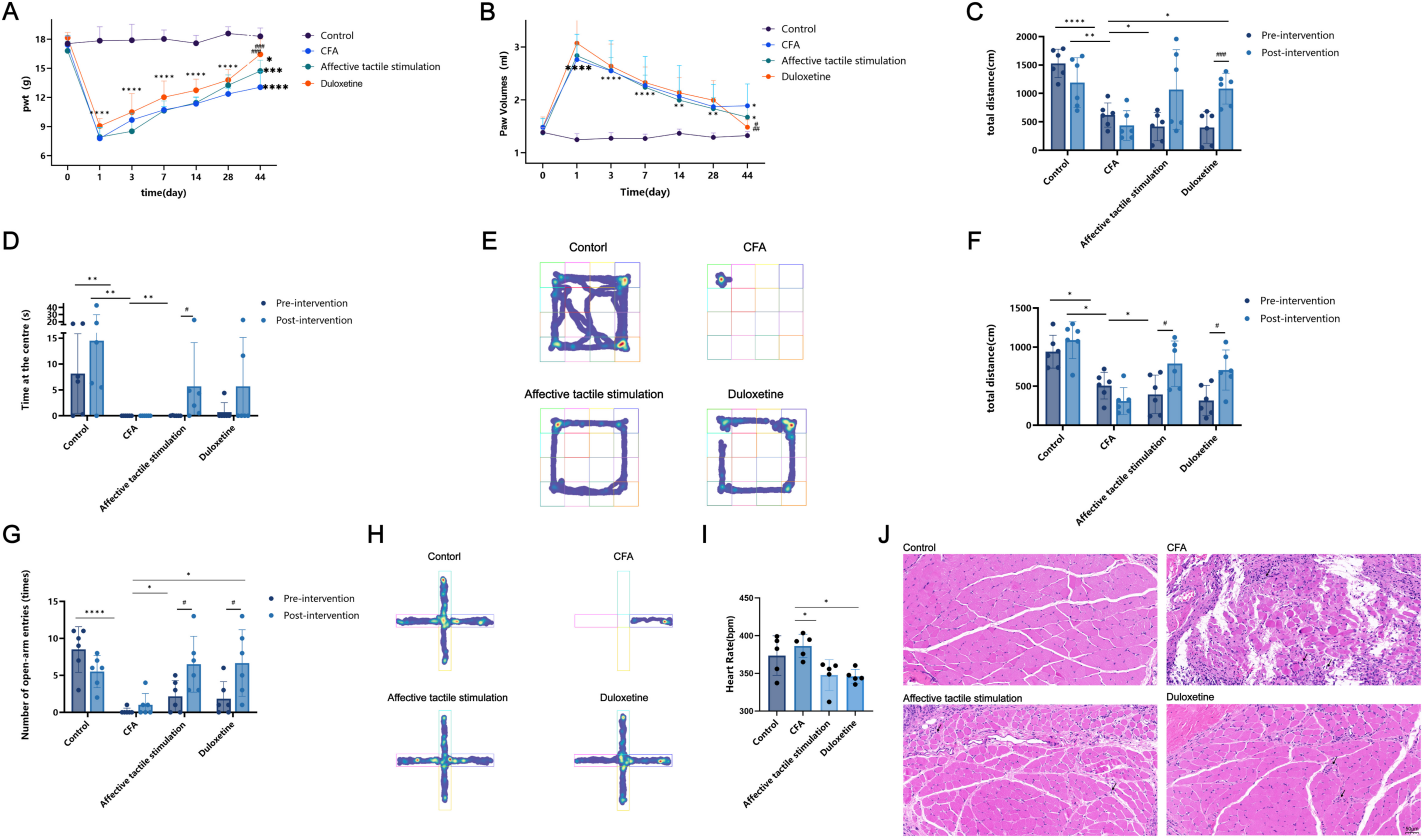
Effects of affective tactile stimulation on pain, anxiety-like behaviors and local inflammation in chronic pain rats. **(A)** Mechanical pain threshold of rats ($\bar{x}$ ± s, *n* = 6) Note: from 1 to 28 days ****P < 0.0001, compared with the control group; 44 days ***P < 0.005, compared with the control group; *P < 0.05, compared with the control group; ^###^*P* < 0.005, compared with the model group. **(B)** Swelling of the toes in rats ($\bar{x}$ ± s, *n* = 6) note: 1–7 days ^****^P < 0.0001, compared with the control group; 14–28 days ^**^P < 0.01, compared with the control group; 44 days *P < 0.05, compared with the control group; ^#^*P* < 0.05, compared with the model group; ^##^*P* < 0.01, compared with the model group. **(C,D)** Open field behavioral results of rats ($\bar{x}$ ± s, *n* = 6) note: ^****^P < 0.001, compared with the control group; ^**^P < 0.01, compared with the model group; *P < 0.05, compared with the model group; ^###^*P* < 0.005, compared with that before the intervention; ^#^*P* < 0.05, compared with that before the intervention. **(E)** Heat map of the open field behavioral trajectory of rats. **(F,G)** Elevated behavioral results of rats ($\bar{x}$ ± s, *n* = 6). ^***^P < 0.001, compared with the control group; *P < 0.05, compared with the control group; *P < 0.05, compared with the model group; ^##^*P* < 0.01, compared with that before the intervention; ^#^*P* < 0.05, compared with that before the intervention. **(H)** Heat map of the cross-elevated behavioral trajectory of rats. **(I)** Heart rate results of rats ($\bar{x}$ ± s, *n* = 5) Note: *P < 0.05, compared with the model group. **(J)** HE staining map of rat feet (×20).

To characterize the therapeutic effects of affective tactile stimulation on the primary sensory and physical manifestations of chronic pain, we first assessed its influence on mechanical pain thresholds and local tissue inflammation. Following CFA induction, animals exhibited a profound and persistent reduction in mechanical pain thresholds beginning on day 1 (*P* < 0.0001), accompanied by significantly increased paw swelling (*P* < 0.005) on days 3, 7, 14, and 28, confirming successful establishment of the chronic inflammatory pain model. After completion of the intervention period (day 44), animals receiving affective tactile stimulation exhibited a preliminary trend toward the attenuation of these peripheral symptoms, characterized by a modest, non-significant increase in mechanical thresholds and a marginal reduction in edema (*P* > 0.05). In contrast, the pharmacological control group (duloxetine) displayed robust and statistically significant elevations in mechanical thresholds (*P* < 0.005) and pronounced reductions in paw swelling (*P* < 0.01). These findings suggest that while affective tactile stimulation may exert a mild modulatory tendency on peripheral nociception, it does not produce the robust immediate attenuation of peripheral inflammation seen with standard pharmacological intervention (Figures 2A,B).

Given that chronic pain markedly suppresses motivational and exploratory behaviors, we next evaluated the ability of affective tactile stimulation to ameliorate affective and exploratory impairments induced by CFA modeling. Behavioral assessment using the open field test demonstrated pronounced behavioral inhibition in the model group, characterized by significant reductions in both total distance traveled (*P* < 0.001) and time spent in the center area (*P* < 0.001) compared to the vehicle control group. Notably, following the 2-week intervention period, both the affective tactile stimulation and duloxetine groups demonstrated significant behavioral recovery relative to the untreated CFA model. Specifically, when compared to the concurrent CFA group, affective tactile stimulation significantly ameliorated the deficit in autonomous exploratory behavior, as evidenced by increased total locomotor distance (*P* < 0.05) and a markedly prolonged duration in the center area (*P* < 0.01). Although within-group comparisons (pre- vs. post-intervention) for total distance did not reach statistical significance in the tactile stimulation group, its post-intervention performance was significantly higher than that of the CFA group. Similarly, the duloxetine group also showed increased total distance traveled (*P* < 0.05) relative to the CFA group. Together, these results suggest that affective tactile stimulation effectively restores behavioral motivation and vitality, demonstrating a therapeutic capacity comparable to standard pharmacological intervention (Figures 2C–E).

To further investigate the psychoregulatory effects of affective tactile stimulation, we assessed its impact on pain-associated anxiety-like behaviors using the elevated plus maze test. Prior to intervention, CFA-treated animals exhibited pronounced anxiety-like phenotypes, characterized by reduced locomotor activity and significantly fewer entries into the open arms compared with the control group. Following the intervention period, affective tactile stimulation showed a marked capacity to restore emotional and exploratory behaviors under anxiogenic conditions. Specifically, affective tactile stimulation significantly increased both total distance traveled and the frequency of open-arm entries (*P* < 0.05), indicating enhanced exploratory motivation and reduced anxiety-like behavior. In contrast, the duloxetine-treated group exhibited a significant increase only in the number of open-arm entries (*P* < 0.05), without a corresponding improvement in overall locomotor activity. Comparative analysis between the two interventions further suggested that affective tactile stimulation produced a broader ameliorative effect on anxiety-related behavioral impairments than pharmacological treatment alone (Figures 2F–H).

To further investigate the physiological basis underlying the affective improvements induced by affective tactile stimulation, heart rate was monitored as an indicator of autonomic cardiovascular regulation and physiological arousal. affective tactile stimulation exhibited a distinct modulatory effect on autonomic function under chronic pain conditions. Specifically, tactile stimulation significantly attenuated pain-associated sympathetic activation, resulting in a markedly lower heart rate in the affective tactile stimulation group compared with untreated CFA animals (*P* < 0.05). In contrast, although duloxetine treatment exhibited a tendency toward reduced heart rate, this effect did not reach statistical significance (*P* > 0.05). These findings suggest that affective tactile stimulation effectively alleviates chronic pain-associated physiological arousal and may contribute to the regulation of autonomic homeostasis during persistent inflammatory pain states (Figure 2I).

Finally, to qualitatively evaluate whether the potential benefits of affective tactile stimulation extend to tissue-level morphology, we conducted histological assessments of the local skeletal muscle. Hematoxylin and eosin (HE) staining of the model group revealed marked pathological damage, including irregular myofibril morphology, swelling, degeneration, blurred striations, nuclear translocation, and extensive deep blue inflammatory cell infiltration within the connective tissue spaces. Based on qualitative visual inspection, affective tactile stimulation appeared to mitigate some of these morphological alterations. While some disorganized and enlarged muscle cells remained, representative tissue sections from the affective tactile stimulation group displayed an apparent reduction in inflammatory cell infiltration and a potential attenuation of pericyte proliferation. This structural presentation was visually comparable to the duloxetine group, which displayed a relatively more regular fiber arrangement and reduced inflammatory infiltrates. Although these qualitative observations preclude definitive conclusions without further quantitative scoring, they visually suggest that affective tactile stimulation may exert localized modulatory effects on peripheral tissue inflammation (Figure 2J).

In conclusion, these findings demonstrate that affective tactile stimulation exerts a profound, bidirectional modulating effect on rats with comorbid chronic pain and anxiety, serving as a powerful regulator of both sensory and affective dimensions of pain. Although its immediate impact on direct peripheral pain thresholds and swelling is secondary to pharmacological intervention, affective tactile stimulation stands out as a uniquely effective therapy for the affective domain—exceling in alleviating anxiety, reclaiming exploratory behavior, and reducing autonomic sympathetic arousal. These broad, systemic benefits are likely mediated by the target activation of dense nerve endings and vascular structures in the cranial region. While the precise neural pathways warrant further elucidation, this study highlights the distinct therapeutic value of affective tactile stimulation as a comprehensive, non-pharmacological strategy for managing chronic pain pathology.

### 3.2 Affective tactile stimulation improves the pathology of chronic pain with anxiety by modulating neural activity in specific areas of the brain after integrating tactile information through the tactile sensory cortex

#### 3.2.1 Effects of affective tactile stimulation on ReHo of FMRI in rats

##### 3.2.1.1 Group main effect results

**FIGURE 3:**
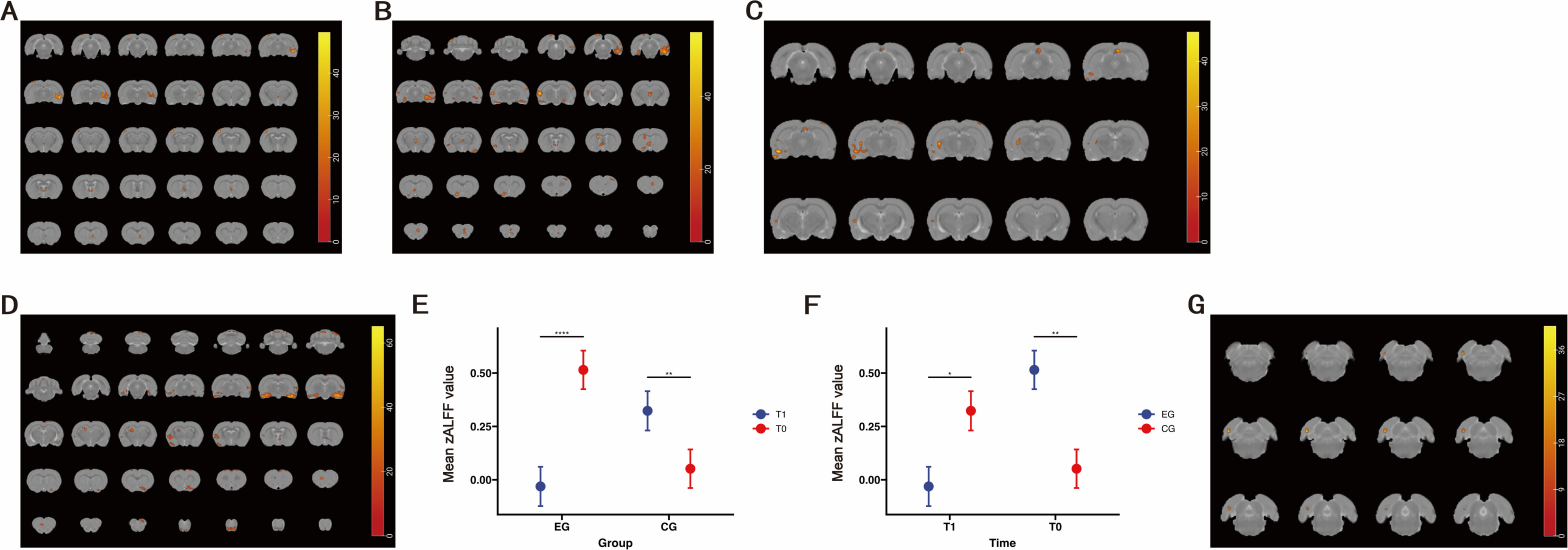
Effects of affective tactile stimulation on ReHo and ALFF of rat FMRI. **(A)** Group main effect of affective tactile stimulation on ReHo of rat FMRI, color bar indicates *F*-value. **(B)** Time main effect of affective tactile stimulation on ReHo of rat FMRI, color bar indicates *F*-value. **(C)** Group main effect of affective tactile stimulation on ALFF of rat FMRI, color bar indicates *F*-value. **(D)** Time main effect of affective tactile stimulation on ALFF effects in rats with FMRI, color bar indicates *F*-value. **(E)** Interaction effect of affective tactile stimulation on ALFF effects in rats with FMRI, results of the fixed-group experiments, dots indicate marginal means, error bar indicates standard error note: CG is the CFA group and EG is the affective tactile stimulation group, T0 is pre-intervention and T1 is post-intervention. ****P < 0.001, Comparison between pre-intervention and post-intervention within the affective tactile stimulation group; **P < 0.01, Comparison between pre-intervention and post-intervention within the CFA group. **(F)** Results of the interaction effect of affective tactile stimulation on ALFF in rat FMRI, results of the fixed-time experiments, dots indicate marginal means, error bar indicates standard error note: CG is the CFA group and EG is the affective tactile stimulation group, T0 is pre-intervention and T1 is post-intervention. *P < 0.05, Comparison between affective tactile stimulation group and CFA group at the pre-intervention; **P < 0.01, Comparison between affective tactile stimulation group and CFA group at the post-intervention. **(G)** Results of the interaction effect of affective tactile stimulation on ALFF in rat FMRI, interaction effect, color bar indicates *F*-value.

Analysis of the ReHo group main effect revealed significant differences between the intervention and CFA groups. Specifically, regional coherence was significantly enhanced in the right hippocampal CA1 area, bilateral lateral septal areas, and left periaqueductal gray (PAG), with the greatest enhancement observed in the hippocampal CA1. Conversely, the regional coherence of the left primary somatosensory cortex barrel layer and left primary visual cortex decreased (Figure 3A). These comparisons indicate that affective tactile stimulation was associated with group dependent differences enhanced local neural synchrony within specific sensory and affective regions compared to the untreated model (Tables 1, 2).

**TABLE 1:** Results of *F*-value test for main effects by group.

Cluster	Brain area	SIGMA peak coordinates			*F*-value	Cluster size
		*X*	*Y*	*Z*		
1	Cornu ammonis 1(R)	6.4	0.02	−5.305	57.54	173
2	Septal region (L)	−0.5	0.92	0.095001	33.58	37
3	Septal region (R)	0.7	0.62	1.295	25.77	22
4	Periaqueductal gray (L)	−0.2	0.02	−3.205	21.21	24
5	Primary somatosensory cortex barrel field (L)	−5	5.12	−1.405	49.34	43
6	Primary visual cortex (L)	−5	6.32	−5.305	45.00	46

(Voxel *P* < 0.001, Cluster *P* < 0.05, GRF corrected).

**TABLE 2:** *T*-test after main effect boosting.

Cluster	Inspection direction	*T*-value	*P*-value
Cluster001	EG-CG	5.04	0.0000
Cluster002	EG-CG	3.14	0.0057
Cluster003	EG-CG	3.40	0.0031
Cluster004	EG-CG	2.56	0.0196
Cluster005	EG-CG	−3.71	0.0016
Cluster006	EG-CG	−2.92	0.0092

CG is the CFA group and EG is the affective tactile stimulation group.

##### 3.2.1.2 Time main effect results

Analysis of the ReHo time main effect to compare pre- and post-intervention values revealed significant differences in several brain regions. Specifically, regional coherence was significantly enhanced in the right medial olfactory cortex, left basal forebrain region, left posterior insula cortex, right periaqueductal gray matter of the midbrain, left striatum, and left medial primary visual cortex. Conversely, there was a significant decrease in regional coherence within the right dentate gyrus, left amygdala-pyriform cortex, left periorbital cortex, right lateral medial olfactory cortex, left primary auditory cortex, right dorsal secondary auditory cortex, left cerebellar molecular layer, and right orbitofrontal regions (Figure 3B). These temporal imaging alterations demonstrate an increase in internal functional coordination within these select brain areas following the intervention (Tables 3, 4).

**TABLE 3:** Results of *F*-value test for time main effect.

Cluster	Brain area	SIGMA peak coordinates			*F-*value	Cluster size
		*X*	*Y*	*Z*		
1	Entorhinal cortex (R)	4.9	−2.68	−1.405	40.12	51
2	Basal forebrain region (L)	−2.3	−1.48	2.195	45.51	112
3	Dentate gyrus (R)	2.8	−1.78	−4.405	30.97	67
4	Amygdalopiriform cortex (L)	−5.6	−1.78	−4.405	43.40	50
5	Perirhinal area 36 (L)	−4.7	−0.58	−6.505	31.62	72
6	Lateral entorhinal cortex external part (R)	6.1	−0.28	−6.805	50.76	475
7	Posterior agralunar insular cortex (L)	−6.2	−0.58	−1.405	25.29	34
8	Basal forebrain region (L)	−0.5	−0.28	−0.205	40.57	106
9	Primary auditory cortex (L)	−6.8	1.52	−4.405	62.60	139
10	Periaqueductal gray (R)	0.1	1.22	−2.605	36.20	99
11	Striatum (L)	−2	3.02	0.095	52.41	54
12	Secondary auditory cortex dorsal Part (R)	6.7	3.62	−4.105	38.98	45
13	Molecular layer of the cerebellum (L)	−4.7	4.52	−11.305	54.62	42
14	Orbitofrontal region (R)	3.1	4.82	3.395	26.00	34
15	Medio lateral secondary visual cortex (L)	−2	6.62	−7.405	44.55	61

Voxel *P* < 0.001, Cluster *P* < 0.05, GRF corrected.

**TABLE 4:** *T*-test results after main effect boosting.

Cluster	Inspection direction	*T*-value	*P*-value
Cluster001	T1-T0	6.52	0.0000
Cluster002	T1-T0	6.54	0.0000
Cluster003	T1-T0	−6.15	0.0000
Cluster004	T1-T0	−8.32	0.0000
Cluster005	T1-T0	−6.19	0.0000
Cluster006	T1-T0	−7.37	0.0000
Cluster007	T1-T0	5.65	0.0000
Cluster008	T1-T0	6.17	0.0000
Cluster009	T1-T0	−7.70	0.0000
Cluster010	T1-T0	6.67	0.0000
Cluster011	T1-T0	6.98	0.0000
Cluster012	T1-T0	−6.68	0.0000
Cluster013	T1-T0	−6.02	0.0000
Cluster014	T1-T0	−5.71	0.0000
Cluster015	T1-T0	6.09	0.0000

T0 is pre-intervention and T1 is post-intervention.

#### 3.2.2 Effects of affective tactile stimulation on the ALFF of FMRI in rats

##### 3.2.2.1 Group main effect results

Analysis of the ALFF group main effect to compare values between the intervention and CFA groups revealed significant regional differences (significance criteria were *P* < 0.001 at the voxel level, *P* < 0.05 at the cluster level, and corrected using GRF at the cluster level). Specifically, the low-frequency amplitude of the left amygdala-pyriform cortex and the left hippocampal CA1 region was significantly enhanced. Conversely, the low-frequency amplitude of the right retrosplenial granular cortex and the right primary visual cortex decreased (Figure 3C). Compared to the model group, the affective tactile stimulation group exhibited enhanced spontaneous neural baseline activity predominantly in these specific functional brain areas (Tables 5, 6).

**TABLE 5:** Results of *F*-value test for group main effect.

Cluster	Brain area	SIGMA peak coordinates			*F*-value	Cluster size
		*X*	*Y*	*Z*		
1	Amygdalopiriform cortex (L)	−7	−2.08	−5.605	38.54	16
2	Cornu ammonis 1 (L)	−6.1	−0.58	−5.605	53.06	106
3	Retrosplenial granular cortex part A (R)	0.5	5.42	−6.505	39.18	36
4	Primary visual cortex binocular area (R)	5	6.32	−5.605	25.62	12

Voxel *P* < 0.001, Cluster *P* < 0.05, GRF corrected.

**TABLE 6:** *T*-test results after main effect boosting.

Cluster	Inspection direction	*T*-value	*P-*value
Cluster001	EG-CG	3.26	0.0044
Cluster002	EG-CG	4.49	0.0003
Cluster003	EG-CG	−3.81	0.0013
Cluster004	EG-CG	−2.38	0.0288

CG is the CFA group and EG is the affective tactile stimulation group.

##### 3.2.2.2 Time main effect results

Analysis of the ALFF time main effect to compare pre- and post-intervention time points revealed significant differences in several brain areas. Specifically, the left entorhinal cortex, right basal forebrain area, left secondary somatosensory cortex, left anterior limbic system, left hippocampal CA3 area, right cerebellar molecular layer, right frontal association cortex, and bilateral primary motor cortices showed a significant increase in low-frequency amplitude. Conversely, the right entorhinal cortex, left subiculum, right primary auditory cortex, and right retrosplenial dysgranular cortex showed a decreasing trend (Figure 3D). Overall, the post-intervention state exhibited a macroscopic functional reorganization of spontaneous neural activity compared to the pre-intervention baseline (Tables 7, 8).

**TABLE 7:** Results of *F*-value test for time main effect.

Cluster	Brain area	SIGMA peak coordinates			*F*-value	Cluster size
		*X*	*Y*	*Z*		
1	Entorhinal cortex (L)	−4.9	−2.68	−1.405	36.49	51
2	Entorhinal cortex (R)	4.4	−2.08	−4.705	79.15	341
3	Subiculum (L)	−4.9	−1.48	−4.705	50.77	299
4	Basal forebrain region (R)	3.2	−2.08	0.695	42.35	90
5	Basal forebrain region (R)	0.5	−0.28	−0.505	36.99	26
6	Secondary somatosensory cortex (L)	−6.7	0.92	−1.705	37.42	126
7	Primary auditory cortex (R)	6.5	1.82	−4.405	43.54	70
8	PreLimbic system (L)	−1	3.02	4.295	45.01	37
9	Cornu ammonis 3 (L)	−2.2	3.62	−2.905	46.43	70
10	Molecular layer of the cerebellum (R)	3.5	4.82	−11.605	37.52	153
11	Frontal association cortex (R)	2	4.82	6.095	40.00	28
12	Primary motor cortex (R)	2.6	6.02	3.395	33.56	66
13	Primary motor cortex (L)	−2.5	6.02	3.395	24.64	27
14	Retosplenial dysgranular cortex (R)	1.4	6.92	−3.505	41.25	48

Voxel *P* < 0.001, Cluster *P* < 0.05, GRF corrected.

**TABLE 8:** *T*-tests after main effect boosts.

Cluster	Inspection direction	*T*-value	*P*-value
Cluster001	T1-T0	6.58	0.0000
Cluster002	T1-T0	−9.67	0.0000
Cluster003	T1-T0	−7.43	0.0000
Cluster004	T1-T0	7.35	0.0000
Cluster005	T1-T0	10.09	0.0000
Cluster006	T1-T0	7.33	0.0000
Cluster007	T1-T0	−8.58	0.0000
Cluster008	T1-T0	7.63	0.0000
Cluster009	T1-T0	10.07	0.0000
Cluster010	T1-T0	−7.83	0.0000
Cluster011	T1-T0	5.55	0.0000
Cluster012	T1-T0	5.43	0.0000
Cluster013	T1-T0	5.38	0.0000
Cluster014	T1-T0	−5.82	0.0000

T0 is pre-intervention and T1 is post-intervention.

##### 3.2.2.3 Interaction effect results

Analysis of the ALFF interaction effect revealed a significant difference in the left perirhinal cortex. *Post hoc* analyses examining the main effects of time and group indicated that within the CFA group, the low-frequency amplitude increased over time, whereas in the affective tactile stimulation group, it showed a significant decrease. Furthermore, when comparing groups at the post-intervention time point, the low-frequency amplitude in the left perirhinal cortex of the affective tactile stimulation group was significantly lower than that of the CFA group (Figures 3E–G). These findings indicate that affective tactile stimulation successfully reverses the intervention-independent elevation of spontaneous neural activity within this specific region (Tables 9–11).

**TABLE 9:** Results of *F*-value test for interaction effects.

Cluster	Brain area	SIGMA peak coordinates			*F*-value	Cluster size
		*X*	*Y*	*Z*		
1	Perirhinal Area 36 (L)	−5.2	2.72	−8.905	58.39	15

Voxel *P* < 0.001, Cluster *P* < 0.05, GRF corrected.

**TABLE 10:** Group fixation test results.

Cluster	Inspection direction	groups	*T*-value	*P*-value
Cluster001	T1-T0	EG	−6.93	0.0000
Cluster001	T1-T0	CG	3.44	0.0029

CG is the CFA group, EG is the affective tactile stimulation group, T0 is pre-intervention and T1 is post-intervention.

**TABLE 11:** Time fixed test results.

Cluster	Inspection direction	Time	*T*-value	*P*-value
Cluster001	EG-CG	T1	−2.72	0.0140
Cluster001	EG-CG	T0	3.62	0.0020

CG is the CFA group, EG is the affective tactile stimulation group, T0 is pre-intervention and T1 is post-intervention.

#### 3.2.3 Affective tactile stimulation effects on whole-brain FC with fMRI in rats

**FIGURE 4:**
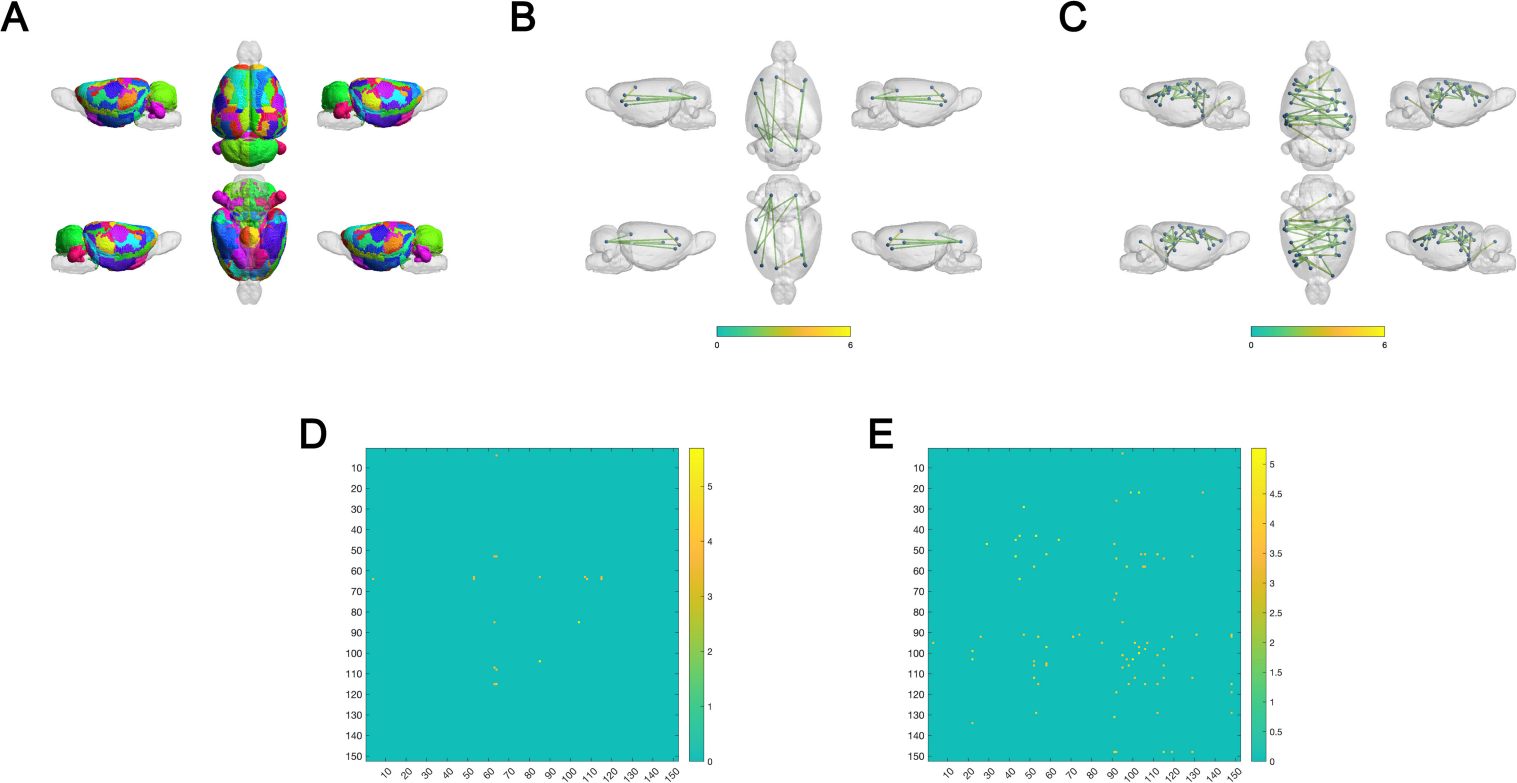
Whole-brain FC results of functional magnetic resonance imaging in rats. **(A)** Schematic of ROIs used for whole-brain FC. **(B)** Spatial distribution of significantly contiguous margins of the post-test over the pre-test, with color bars denoting the *F*-values. **(C)** Spatial distribution of significantly contiguous margins of the pre-test over the post-test, with color bars denoting the *F*-values. **(D)** *T*-value plots of significantly contiguous margins of the post-test over the pre-test, with color bars denoting the *t*-values, Note: x-axis = ROI (Region of Interest) number; y-axis = ROI number. **(E)** *T*-value plots of significantly contiguous margins of the pre-test over the post-test, with color bars denoting the *t*-values. x-axis, ROI number; y-axis, ROI number.

Whole-brain functional connectivity (FC) was analyzed using fMRI. While no significant components were identified for group main effects or interaction effects, significant main effects of time were observed (Figure 4A). There were no significant components for the group main effects and interaction effects, and the following statistical results were obtained for the main effects of time. The results of the posttest over the pretest showed that the *P*-value of the component calculated with 5,000 permutations was 0.02536. There were nine contiguous edges within the component, and the ROIs and *t*-values corresponding to the contiguous edges were as follows (see Figures 4B,D and Table 12). The results of the pre-test over the post-test showed that the *P*-value of the component calculated with 5,000 substitutions was 0.0006. There were 40 connecting edges within the component, and the ROIs and T-values corresponding to the connecting edges were as follows (see Figures 4C,E and Table 13). These results suggest that brain functional connectivity was significantly altered between the pre- and post-tests of the affective tactile stimulation intervention, as evidenced by a significant increase in the connectivity strength of a smaller visual-spatial-emotional integration network (9 edges), which conferred emotional significance to the environment and enhanced environmental vigilance, accompanied by a significant decrease in the connectivity strength and neural alertness of a larger, more complex network involving spatial navigation, situational memory, multimodal integration, and behavioral modulation (40 edges) exhibited a significant decrease in connectivity strength and neural alertness. This weaker connectivity was accompanied by increased neural efficiency and a reduced need for environmental threat assessment.

**TABLE 12:** Significantly higher connecting edge information for post-test than pre-test.

ROI1	ROI2	*T*
Secondary visual cortex (R)	Cerebellar molecular layer (L)	3.75
Ungranular guiding lobe cortex (R)	Cerebellar molecular layer (R)	3.70
Secondary visual cortex (L)	Cerebellar molecular layer (R)	3.81
Cerebellar molecular layer (L)	Anterior limbic system (L)	4.02
Anterior limbic system (L)	Primary somatosensory cortex forelimb (R)	5.69
Cerebellar molecular layer (L)	Primary somatosensory cortex jaw (L)	4.19
Cerebellar molecular layer (R)	Primary somatosensory cortex jaw (R)	4.04
Cerebellar molecular layer (L)	Primary somatosensory cortex upper lip (L)	3.66
Cerebellar molecular layer (R)	Primary somatosensory cortex upper lip (L)	4.25

**TABLE 13:** Pre-test over post-test significant connecting edge information.

ROI1	ROI2	*T*
Intermediate pedunculopontine nucleus (L)	Inside the internal olfactory cortex (L)	4.68
Gray matter bundle (L)	Lateral cortex of the internal olfactory area (L)	4.96
Interparietal nucleus (L)	Secondary visual cortex (L)	5.27
Lateral parietal association cortex (R)	Medial parietal association cortex (R)	4.37
Lateral inner olfactory cortex inner (L)	Cerebellar molecular layer (R)	4.95
Lateral cortex of the internal olfactory region (L)	Primary auditory cortex (L)	4.03
Lateral posterior parietal cortex of the mouth (R)	Primary auditory cortex (L)	3.74
Lateral olfactory area cortex (R)	Primary auditory cortex (R)	3.88
Secondary visual cortex (R)	Primary auditory cortex (R)	4.33
Posterior dorsal parietal cortex (L)	Primary auditory cortex (R)	4.08
Insula without granule cells cortex (L)	Primary motor cortex (L)	3.85
Prelimbic cortical system (L)	Primary motor cortex (L)	3.85
Medial parietal association cortex (R)	Primary somatosensory cortex, barrel area L	4.46
Dorsolateral orbitofacial cortex (R)	Primary somatosensory cortex, dysgranular cell area L	4.49
Primary somatosensory cortex (L)	Primary somatosensory cortex, maladaptive granule cell area L	4.34
Dorsolateral orbitoventral cortex (R)	Primary somatosensory cortex forelimb (L)	5.13
Primary somatosensory cortex barrel region (L)	Primary somatosensory cortex forelimb (L)	3.97
Primary sensory cortex maladaptive granule cell area (R)	Primary somatosensory cortex forelimb (L)	5.00
Lateral parietal association cortex (R)	Primary somatosensory cortex forelimb (R)	3.64
Secondary visual cortex (R)	Primary auditory cortex (R)	3.87
Posterior dorsal parietal cortex (L)	Primary auditory cortex (R)	3.70
Medial combined parietal cortex (R)	Primary sensory cortex hindlimb (L)	3.62
Lateral combined parietal cortex (R)	Primary sensory cortex hindlimb (R)	4.06
Medial combined parietal cortex (R)	Primary sensory cortex hindlimb (R)	3.71
Primary sensory cortex barrel area (R)	Primary sensory cortex hind limb (R)	4.41
Primary motor cortex (L)	Primary sensory cortex jaw (L)	3.76
Lateral parietal coalition cortex (R)	Primary sensory cortex shoulder (R)	3.66
Primary sensory cortex, maladaptive granule cell area (L)	Primary sensory cortex shoulder (R)	3.95
Lateral secondary visual cortex (R)	Primary sensory cortex—upper lip (L)	4.01
Primary sensory cortex barrel area (R)	Primary sensory cortex—upper lip (L)	3.70
Primary sensory cortex hindlimb area R	Primary sensory cortex—upper lip L	4.38
Primary sensory cortex shoulder (R)	Primary sensory cortex—upper lip L	4.05
Primary auditory cortex (R)	Primary visual cortex (L)	3.71
Lateral secondary visual cortex (L)	Secondary auditory cortex, dorsolateral area L	4.17
Primary sensory cortex shoulder (R)	Secondary auditory cortex, dorsolateral area L	3.67
Primary auditory cortex (L)	Secondary auditory cortex, ventral region (L)	4.16
Dorsolateral orbital cortex (R)	Anterior cingulate gyrus (R)	3.85
Primary auditory cortex (L)	Temporal lobe association cortex (R)	4.21
Primary auditory cortex (R)	Temporal lobe association cortex (R)	3.63
Primary Sensory Cortex Lower Lip Region (L)	Temporal lobe association cortex (L)	3.73

Collectively, affective tactile stimulation, after integrating tactile information through tactile stimulation of the sensory cortex by the head, modulates neural activity in specific areas of the brain, especially the coherence and low-frequency amplitude of neural activity in areas such as the hippocampal CA1 area, entorhinal cortex, and septal areas, and remodels the cerebellum-sensory cortex-entorhinal cortex-hippocampus functional connectivity network, thus improving the pathology of chronic pain with anxiety. The hippocampal CA1 and periorbital cortex may be key regulatory targets.

#### 3.2.4 CA1 activity may contribute to tactile stimulation related behavioral modulation

To establish a stable baseline for subsequent circuit-specific manipulations, we first verified the successful establishment of the chronic inflammatory pain model accompanied by anxiety-like behaviors. Behavioral assessments prior to intervention demonstrated marked behavioral suppression in CFA-treated animals, characterized by significantly reduced total distance traveled in the open field test (OFT) compared with control animals. Concurrently, the model group exhibited a significantly decreased paw withdrawal threshold (PWT), confirming persistent mechanical hypersensitivity. Importantly, no significant differences were detected among the designated experimental groups before intervention, indicating comparable baseline conditions for subsequent evaluation of tactile and optogenetic manipulations.

**FIGURE 5:**
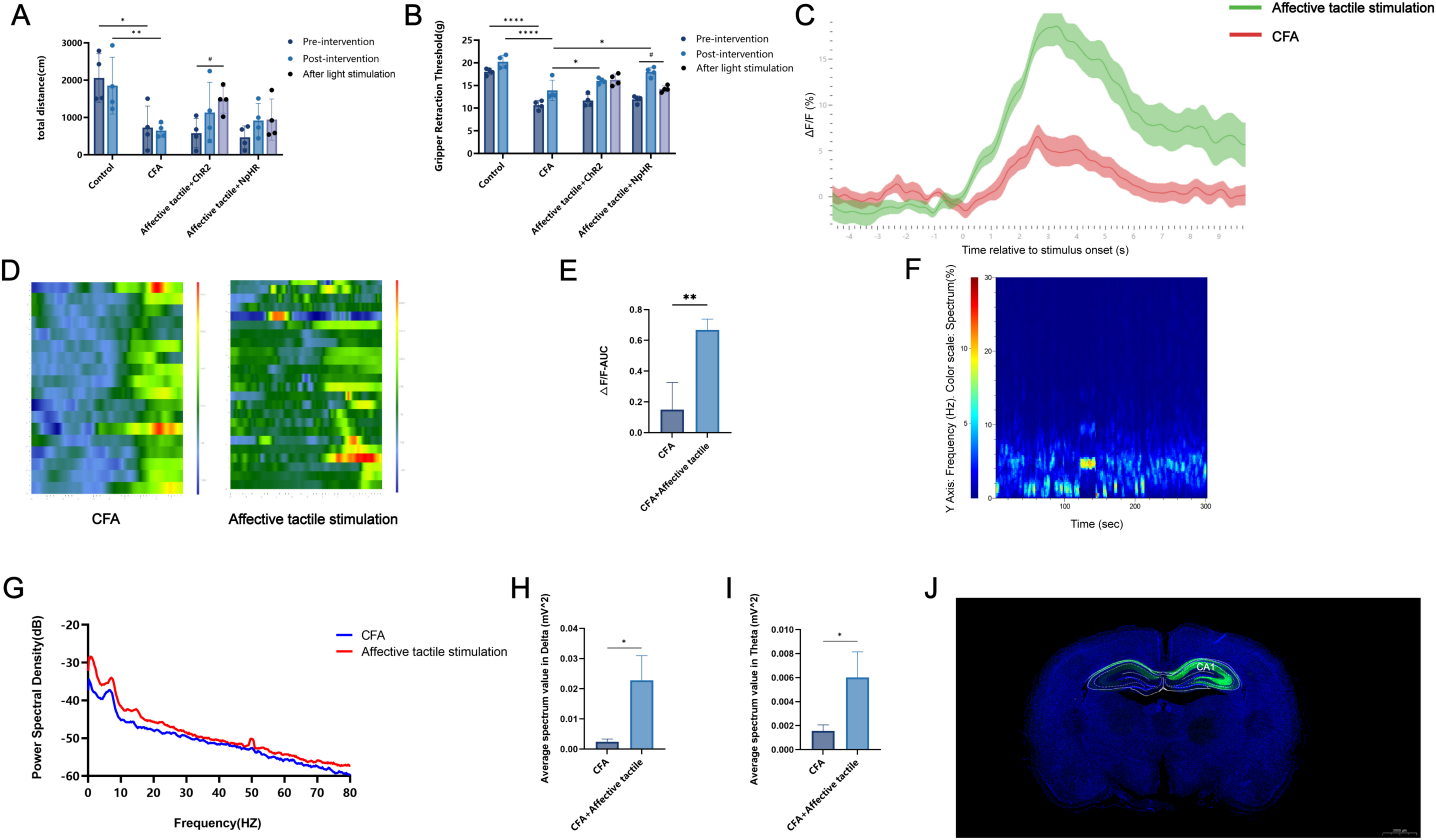
CA1 activity is associated with pain and anxiety related behavioral changes after affective tactile stimulation, rat CA1 spike potential results ($\bar{x}$ ± s, *n* = 4). **(A)** Rat absenteeism behavior ($\bar{x}$ ± s, *n* = 4). **P < 0.01, compared with CFA group; *P < 0.05, compared with CFA group; *P < 0.05, compared with pre-intervention. **(B)** Rat pain threshold ($\bar{x}$ ± s, *n* = 4). ****P < 0.0001, compared with CFA group; *P < 0.05, compared with CFA group;*P < 0.05, compared with pre-intervention. **(C)** Plot of the mean calcium signal (ΔF/F). Solid lines represent the mean ΔF/F (%), and shaded areas indicate ± SEM. **(D)** Heat map of the mean change in calcium signal (ΔF/F). **(E)** Plot of the area under the calcium signal (ΔF/F) curve for intergroup comparison. **P < 0.01, compared with the CFA group. **(F)** Time-frequency plot of LFP in the affective tactile stimulation group. **(G)** Comparison of PSD. **(H,I)** Comparison of mean spectral values of delta and theta bands. *P < 0.05, compared with the CFA group. **(J)** Representative histological image of viral expression in the hippocampus. Coronal brain section demonstrating the expression of the viral vector (green) in the target hippocampal CA1 region. The area delineated by white lines indicates the primary injection and recording/stimulation site within the dorsal hippocampus. Cell nuclei were counterstained with DAPI (blue). Scale bar: 2,000 μm.

To further investigate the potential neural correlates mechanisms underlying the therapeutic effects of affective tactile stimulation, tactile intervention was combined with *in vivo* optogenetic modulation of hippocampal CA1-related neural circuits. Following the intervention period, affective tactile stimulation alone significantly increased PWT compared with the untreated model group (*P* < 0.05), indicating partial alleviation of mechanical hypersensitivity. Moreover, when affective tactile stimulation was paired with optogenetic activation of the targeted neural circuits, animals exhibited significant recovery of spontaneous exploratory behavior, as reflected by increased total distance traveled in the OFT relative to pre-intervention levels (*P* < 0.05). In contrast, optogenetic inhibition of these circuits failed to improve locomotor or exploratory activity (*P* > 0.05). Interestingly, although circuit activation did not further enhance the tactile-induced elevation in PWT, optogenetic inhibition resulted in a significant increase in nociceptive threshold compared with the pre-stimulation state (*P* < 0.05). Collectively, these observations demonstrate that ChR2-mediated activation and eNpHR-mediated inhibition of the CA1 circuit produce divergent effects across different behavioral metrics (Figures 5A,B).

Given the established involvement of the hippocampus in affective pain processing, we next employed *in vivo* fiber photometry to monitor neuronal calcium dynamics (ΔF/F) within the CA1 region during affective tactile stimulation intervention. Affective tactile stimulation was temporally associated with pronounced activation of local neuronal populations in the hippocampal CA1 area. Real-time calcium signal traces indicated revealed that animals receiving affective tactile stimulation exhibited consistently elevated calcium transient amplitudes compared with the CFA group, followed by a gradual return to baseline after peak activation (Figure 5C). This enhanced neuronal activity was further supported by heatmap analyses, which demonstrated increased overall calcium signal intensity during the affective tactile stimulation intervention relative to the model condition (Figure 5D). Quantitative analysis further corroborated these observations, showing that the area under the curve (AUC) of the mean calcium signals was significantly greater in the affective tactile stimulation group than in untreated CFA (*P* < 0.01; Figure 5E). Collectively, these findings suggest that affective tactile stimulation is accompanied by dynamic changes in hippocampal CA1 neuronal activity, highlighting a potential correlative link between this intervention and neural processes associated with affective regulation during chronic pain states.

#### 3.2.5 CA1 local field potential results

Local field potential results: The time-frequency distribution of LFP in hippocampal CA1 during affective tactile stimulation intervention, from the time-frequency plot, showed that the changes in the affective tactile stimulation group were concentrated in the low-frequency range. This suggests that the affective tactile stimulation intervention may have enhanced the oscillatory strength of certain low-frequency bands (e.g., delta and theta bands) in hippocampal CA1 (Figure 5F). The power spectral density (PSD) results showed that the affective tactile stimulation group had higher power values in the delta and theta bands than the CFA group (Figure 5G). A comparison of power in the theta band (4–8 Hz) and power in the delta band (1–4 Hz) was carried out (Figures 5H,I). As can be seen from the bar graphs, the average power in both the theta and delta bands in the affective tactile stimulation group was significantly higher than that of the CFA group and was statistically significant (*P* < 0.05). This suggests that the affective tactile stimulation intervention showed significant enhancement in low-frequency oscillations (especially in the delta and theta bands). In conclusion, affective tactile intervention can significantly increase the power level of low-frequency oscillations in the hippocampal CA1 region and enhance LFP activity in theta and delta bands. Representative viral expression and the primary recording/stimulation site in the dorsal hippocampal CA1 region are shown (Figure 5J).

#### 3.2.6 CA1 functional connectivity results

##### 3.2.6.1 Group main effect results

**FIGURE 6:**
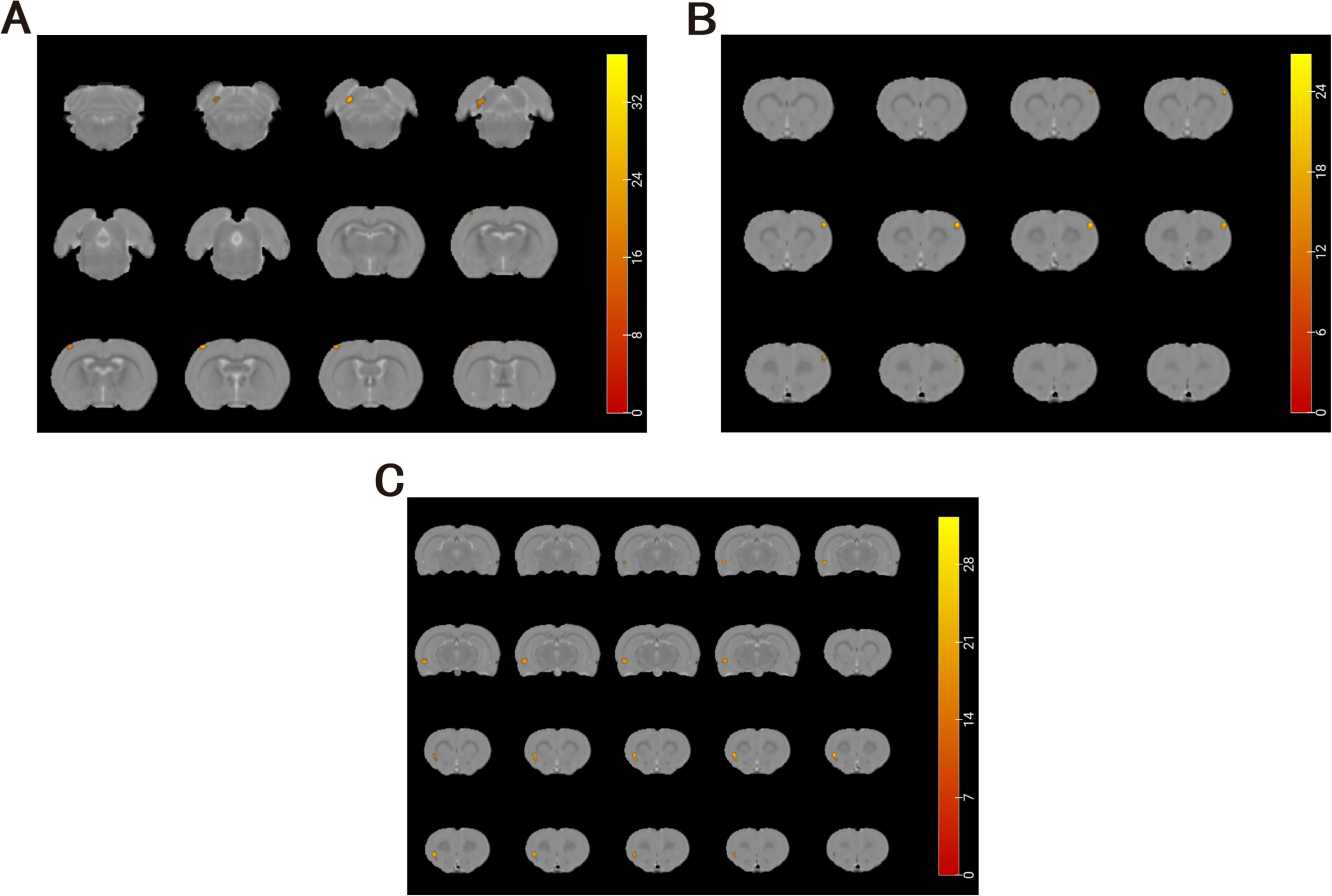
CA1 functional connectivity and inner olfactory cortex functional connectivity. **(A)** Group main effect of CA1 functional connectivity outcome; the color bar indicates the *F*-value. **(B)** Time main effect on CA1 functional connectivity outcome. The color bar indicates the *F*-value. **(C)** Time main effect of entorhinal cortex functional connectivity; the color bar indicates the *F*-value.

Functional connectivity between the two groups was compared using ANOVA, and the results showed significant differences in brain regions: hippocampal CA1 connectivity with the left cerebellar molecular region and the left first somatosensory cortex oligomeric granule cell region was significantly elevated compared to the CFA group (Figure 6A and Tables 14, 15).

**TABLE 14:** Results of *F*-value test for main effects by group.

Cluster	Brain area	SIGMA peak coordinates			*F*-value	Cluster size
		*X*	*Y*	*Z*		
1	Cerebellar molecular layer (L)	−3.1	3.02	−8.91	33.66	26
2	Primary sensory cortex, less granular cell area (L)	−4.9	5.72	−0.50	42.51	19

Voxel *P* < 0.001, Cluster *P* < 0.05, GRF corrected.

**TABLE 15:** *T*-test after main effect boosting.

Cluster	Inspection direction	*T*-value	*P-*value
Cluster001	EG-CG	3.58	0.0020
Cluster002	EG-CG	3.40	0.0300

CG is the CFA group and EG is the affective tactile stimulation group.

##### 3.2.6.2 Time main effect results

Pre- and post-functional connectivity were compared using ANOVA analyses (significance criteria were *P* < 0.001 at the voxel level, *P* < 0.05 at the cluster level, and corrected using GRF at the clump level), which showed a significant decrease in the brain region where there was a significant difference: hippocampal CA1, in contrast to the right first somatosensory cortical jaw region (Figure 6B and Tables 16, 17).

**TABLE 16:** Results of *F*-value test for time main effect.

Cluster	Brain area	SIGMA peak coordinates			*F*-value	Cluster size
		*X*	*Y*	*Z*		
1	Primary sensory cortex, maxillary region(R)	5.3	4.22	2.80	31.00	13

Voxel *P* < 0.001, Cluster *P* < 0.05, GRF corrected.

**TABLE 17:** *T*-test after main effect boosting.

Cluster	Inspection direction	*T*-value	*P*-value
Cluster001	T1-T0	−5.71	0.0000

T0 is pre-intervention and T1 is post-intervention.

##### 3.2.6.3 Interaction effect results

No significant results.

#### 3.2.7 Functional connectivity of the internal olfactory cortex

##### 3.2.7.1 Group main effect results

No significant results.

##### 3.2.7.2 Time main effect results

Pre- and post-functional connectivity were compared using ANOVA analyses (significance criteria were *P* < 0.001 at the voxel level, *P* < 0.05 at the cluster level, and corrected using GRF at the cluster level), and the results showed that the brain regions with significant differences: the inner olfactory cortex appeared to be significantly elevated in its connectivity to the right hippocampal CA1 region and to the left granulomatous insular cortex (Figure 6C and Tables 18, 19).

**TABLE 18:** Results of *F*-value test for time main effect.

Cluster	Brain area	SIGMA peak coordinates			*F*-value	Cluster size
		*X*	*Y*	*Z*		
1	Cornu Ammonis 1 (L)	−6.10	0.02	−5.01	25.68	23
2	Ungranulated insular cortex (L)	−4.60	1.22	2.80	37.51	21

Voxel *P* < 0.001, Cluster *P* < 0.05, GRF corrected.

**TABLE 19:** *T*-test after main effect boosting.

Cluster	Inspection direction	*T*-value	*P*-value
Cluster001	T1-T0	4.99	0.0000
Cluster002	T1-T0	6.33	0.0000

T0 is pre-intervention and T1 is post-intervention.

##### 3.2.7.3 Interaction effect results

No significant results.

## 4 Discussion

In the open-field test conducted in this study, the total distance traveled by the CFA model group decreased significantly. This not only reflects movement avoidance caused by chronic pain but also indicates a reduction in exploratory motivation resulting from a depression-like state. In contrast, the affective tactile stimulation intervention significantly restored the total distance traveled. This indicates that the intervention not only alleviated noxious sensations but also reversed the chronic pain-induced movement inhibition and diminished exploratory drive, demonstrating its comprehensive regulatory role at the emotional and motivational levels. These behavioral improvements are likely driven by the anatomical and physiological characteristics of the stimulated area. The head is rich in nerve endings and blood vessels; affective tactile stimulation applied to this region effectively stimulates these nerve endings, promoting the release of neurotransmitters closely related to emotion regulation, such as dopamine and serotonin. Concurrently, the biomechanical effects of touch improve peripheral blood flow and microcirculation, increasing cerebral blood supply to enhance brain function and affect the mood (Liu et al., 2022; Case et al., 2023). Ultimately, this intervention exerts a synergistic, two-way regulatory effect. At the peripheral level, the biomechanical properties of affective tactile stimulation improve microcirculation and blood flow, contributing directly to anti-inflammatory and analgesic outcomes. At the central level, it modulates sensory-affective processing and regulates anxiety-like behaviors. By bridging these two domains, the intervention achieves a comprehensive, bidirectional modulation of chronic pain and associated emotional distress.

Merkel cells (Woo et al., 2014) and tactile vesicles (Bajwa and Al, 2025) are the first to be activated when a gentle physical stimulus reaches the skin surface. These mechanoreceptors convert the physical stimulus into a bioelectrical signal that is transmitted via Aβ (Sehlstedt et al., 2016) and C nerve fibers (McGlone et al., 2012). The signals are transmitted to the trigeminal ganglion via the supramarginal nerve (Bhuiyan et al., 2023), and subsequently, at the brainstem stage (Ebert et al., 2021), the tactile signals complete their spatial localization in the trigeminal sensory principal nucleus, and some of the cross-fibers project to the thalamus via the trigeminal thalamus tract, where they are finely encoded and then uploaded to the primary somatosensory cortex in the lower part of the postcentral gyrus (Glassman, 1994).

Beyond simple sensory processing, regional coherence (ReHo) analysis reveals that affective tactile stimulation exerts a profound neural integration effect. The significant elevation of ReHo values within the default mode network (DMN), alongside enhanced synchrony in the right hippocampus and bilateral septal regions, suggests that the intervention promotes emotional stabilization and memory remodeling. The hippocampus is the brain’s key region for processing emotions (Ma et al., 2021; Liu et al., 2023). We propose that the increased ReHo in the hippocampus—a nexus for emotional processing—reflects structural plasticity, such as increased dendritic spine density or the integration of newborn neurons, which directly supports memory refinement in chronic pain states. This suggests that affective tactile stimulation may optimizes the spatiotemporal coding efficiency of neuronal ensembles by fine-tuning local inhibitory microcircuits. Simultaneously, a reduction in primary sensory and visual cortex sums, as well as a significant enhancement of periaqueductal gray matter in the midbrain in the temporal dimension, was highly correlated with a decrease in threshold scores for the pain dimension, suggesting that the intervention was effective in correcting the abnormalities of cross-modal sensory integration. This “bi-directional modulation”—characterized by strengthened cognitive synchrony and suppressed pain-affective network activity—provides robust functional imaging evidence for the therapeutic efficacy of touch.

Dynamic changes in low-frequency amplitude (ALFF) reveal the multilevel effects of affective tactile stimulation on neuronal metabolic activity, and analysis of the results suggests that affective tactile stimulation regulates the pain-emotion network through a dual mechanism: on the one hand, activation of the amygdala and hippocampal CA1 promotes pleasurable emotions through the limbic reward system; on the other hand, inhibition of the posterior granule cells of the pressor a-cortex and the primary visual cortex blocks the pain-emotion vicious cycle of assessment. The enhancement of amygdala pyriform cortex and hippocampal CA1 activity may be associated with enhanced glutamatergic transmission or inhibition of γ-aminobutyric acid receptor expression (Ji et al., 2010; McDonald and Zaric, 2015; Wu et al., 2024). Further comparisons revealed that the magnitude of ALFF changes in emotion-related brain regions was significantly higher than that before the intervention in terms of temporal differences, whereas the suppression of more affected activity in the cerebellar molecular layer and secondary sensory cortex revealed the unique modulatory advantage of the head intervention on the central emotional network. Meanwhile, the left internal olfactory cortex, an important nucleus for the cognitive processing of emotions and memories (Knierim et al., 2014), especially in terms of functional connectivity with the hippocampus (Jiang et al., 2022), reflected significant differences at both the level of fixation groups and time.

Dynamic changes in functional connectivity (FC) further revealed the hierarchical modulation of neural circuits by affective tactile stimulation. It was found that the strength of the left limbic system-left cerebellar molecular layer and primary somatosensory cortex connections was significantly enhanced after the intervention, whereas the left intermediate pedunculopontine nucleus-left internal olfactory cortex internal connections showed a tendency to weaken. The former may inhibit the generalization of pain-related negative emotions by enhancing the cognitive control of the limbic system and modulating the excitability of the pyramidal neurons (Zhao et al., 2022); the latter interpeduncular nucleus may alleviate the nociceptive hypersensitivity state by blocking the abnormal transmission of dopaminergic signals to the internal olfactory cortex (McLaughlin et al., 2017). Meanwhile, the significantly elevated connectivity strength of bilateral cerebellar molecular layer with different motor cortex may block the enhancement of pain-emotion interaction by inhibiting the abnormal cross-modal sensory integration. This suggests that affective tactile stimulation not only modulates emotional networks, but also inhibits the central transmission of peripheral pain signals.

Together, these imaging findings suggest that tactile stimulation is associated with functional reorganization of brain networks involved in sensory, affective, and pain-related processing. Although indirect imaging measures such as ReHo, ALFF, and FC cannot directly demonstrate synaptic plasticity, neurotransmitter changes, memory remodeling, or descending pain inhibition, they provide a crucial network-level foundation for subsequent targeted analyses. Specifically, the consistent involvement of the CA1 region in both regional activity and connectivity analyses suggested that it may be an important node associated with tactile-stimulation-related behavioral improvement. To bridge this macroscopic evidence with local circuit mechanisms, we therefore further examined CA1 neuronal activity and oscillatory dynamics using fiber photometry and LFP recordings.

In alignment with this network-level hypothesis, our localized recordings revealed that the calcium signal (ΔF/F) and low-frequency oscillatory power in the theta and delta frequency bands in the hippocampal CA1 area were significantly enhanced after the affective tactile stimulation intervention compared with the CFA group. Importantly, these enhanced neural dynamics were directly correlated with the improvement of anxiety behaviors. This result was highly consistent with behavioral findings, suggesting that enhanced neuronal activity in the CA1 area may be the key neural basis for affective tactile stimulation to relieve pain and anxiety. Significantly elevated calcium signals in area CA1 indicated enhanced neural activity in this region. Theta oscillations are a central feature of hippocampus-dependent memory integration and emotion regulation (Lansink et al., 2016; Li et al., 2024). It has been demonstrated that enhanced theta rhythm reverses memory deficits in brain-injured animals (Broussard et al., 2023), delta oscillations are associated with neuroplasticity in pain perception (Rutishauser et al., 2010), and both theta activity and delta inhibition are correlated with anxiety phenotypes (Engin et al., 2016; Wang et al., 2024). The optogenetic results confirm that affective tactile stimulation alleviates anxiety by modulating neural oscillations in the hippocampal CA1, a critical hub in the pain-anxiety continuum. Notably, this circuit exhibits a distinct functional asymmetry: while excitatory modulation ameliorates specific exploratory deficits, inhibitory interventions fail to exert a reciprocal effect across all behavioral metrics, pointing to inherent compensatory mechanisms or network redundancy. Ultimately, these findings reveal that affective tactile stimulation operates not only by reshaping affective networks but also by actively gating the central transmission of peripheral nociceptive signals.

According to the functional connectivity analysis of the internal olfactory cortex, the functional connectivity between the internal olfactory cortex and the CA1 region of the hippocampus was significantly enhanced after the affective tactile stimulation intervention was applied, this suggests that the CA1-inner olfactory cortex loop may be a central hub in the affective tactile stimulation modulation of chronic pain with anxiety. The entorhinal olfactory cortex, as the main source of information input to CA1 (Bell et al., 2021), is responsible for integrating spatial, sensory, and emotional information (Canto et al., 2008), which is encoded into long-term memory or emotional states by theta oscillations in the CA1 region. In the present study, the theta power of CA1 was enhanced after the affective tactile stimulation intervention, which may facilitate the “decoupling” of pain signals and anxiety by enhancing synaptic plasticity from the olfactory cortex to CA1. It has been shown that the olfactory cortex over-transmits injurious signals to CA1, leading to the solidification of pain memory and anxiety behaviors (Lyu et al., 2013), whereas affective tactile stimulation may inhibit the abnormal input from the olfactory cortex, restoring the dynamic balance of pain-emotion information in CA1. This mechanism is highly consistent with previous behavioral findings, including elevated pain threshold and prolonged dwell time in the elevated open arm, and reveals the key role of the “peripheral stimulus-inner olfactory cortex-CA1 loop” in improving mood.

Meanwhile, the functional connectivity between the CA1 area of the hippocampus and the molecular layer of the left cerebellum was significantly enhanced after the affective tactile stimulation intervention, suggesting that the cerebellum may be involved in the integration of pain-emotion regulation through the cerebello-thalamic-hippampal pathway. The cerebellar molecular layer is an important input layer to the cerebellar cortex (Lehre and Danbolt, 1998), and its granule cells receive projections from the peripheral somatosensory receptors (Jin et al., 2016) and spinal cord (Edvinsson et al., 2011). Abnormal activation of the cerebellar molecular layer may lead to motor coordination deficits and nociceptive sensitization, whereas affective tactile stimulation enhances the functional connectivity between CA1 and the cerebellar molecular layer, which may re-code pain signals as “modifiable” information through the synchronization of theta and delta oscillations and inhibit cerebellar over-responsiveness to abnormal sensory inputs. The functional connectivity between CA1 and the left primary somatosensory cortex (S1) was significantly enhanced, suggesting that S1 may be involved in the central processing of pain signals through the “CA1-S1” loop. S1, as a primary integrator of somatosensory information (Tamè et al., 2016), is often overactivated and cortically reorganized in chronic pain (Bushnell et al., 1999; Takeda et al., 2022), leading to nociceptive sensitization and negative emotional reinforcement.

This study provides the first comprehensive evidence that affective tactile stimulation modulates the neural oscillations and functional connectivity of the hippocampal CA1 to resolve the comorbidities of chronic pain and anxiety. As a critical integrative node, CA1 facilitates the integration of tactile sensory inputs into broader affective networks, offering a transformative perspective on non-pharmacological interventions and positioning tactile stimulation as a sophisticated tool for neural circuit recalibration.

While this study provides valuable insights, certain methodological constraints warrant consideration. Our assessment of peripheral inflammation was largely qualitative and based on representative histological sections. Definitively confirming these structural trends will require future investigations utilizing rigorous quantitative techniques, including morphometric analysis and blinded histological scoring. Furthermore, the absence of independent optogenetic controls, namely light-only and virus-only cohorts, means we cannot entirely rule out potential non-specific artifacts arising from sustained illumination or viral transduction. Given this experimental constraint, the optogenetic results are most appropriately interpreted as indicating a partial modulatory influence rather than a strict causal mechanism.

Additionally, the fMRI findings suggested that the right CA1 was involved in tactile stimulation related brain activity changes. Therefore, subsequent invasive recordings focused primarily on the right CA1. However, because viral manipulation was bilateral and the study was not specifically designed to compare left versus right CA1 function, the present data should not be interpreted as direct evidence for hemispheric lateralization.

While our functional imaging findings (ReHo and ALFF) indicate alterations in regional spontaneous activity and functional connectivity patterns associated with affective tactile stimulation. ReHo, ALFF, and FC are indirect measures and cannot determine synaptic plasticity, neurotransmitter mechanisms, or causal circuit directionality. Therefore, interpretations linking these specific macroscopic imaging alterations to cognitive remodeling remain speculative. Future investigations incorporating complementary molecular techniques, circuit-level validations, and targeted cognitive-behavioral paradigms are essential to definitively substantiate these hypothesized neural mechanisms.

These findings establish a critical foundational role for overall CA1 regional activity and demonstrate altered connectivity concurrent with behavioral improvements, the exact neural architectures remain to be fully elucidated. Future investigations are highly warranted to dissect the precise local microcircuits using cell-type-specific promoters (e.g., targeting distinct interneuron subtypes or pyramidal lineages), as well as to map the broader polysynaptic anatomical pathways through advanced circuit-tracing studies.

## Data Availability

The original contributions presented in the study are included in the article/Supplementary material, further inquiries can be directed to the corresponding author.

## Supplementary material

Supplementary Figure 1 Part of the experimental apparatus. Affective tactile stimulator.

Supplementary Figure 2 Rat photosensitive protein injection. **(A)** Schematic diagram of rat photosensitive protein injection. **(B)** Frozen-section images of photosensitive protein injection sites.

Supplementary Figure 3 Rat calcium signaling virus injection. **(A)** Schematic diagram of rat calcium signaling virus injection. **(B)** Frozen-section images of calcium signaling virus injection sites.
